# Tribal women empowerment through entrepreneurship: evidence from Mayurbhanj District, Odisha

**DOI:** 10.3389/fsoc.2023.1158770

**Published:** 2023-08-03

**Authors:** S. Naveen, Jayanta Kumar Parida, Itishree Panda

**Affiliations:** School of Social, Financial and Human Sciences (KSFH), Kalinga Institute of Industrial Technology (KIIT) Deemed to be University, Bhubaneswar, Odisha, India

**Keywords:** entrepreneurship, women, tribal, Women Empowerment Index, empowerment

## Abstract

**Introduction:**

Empowering women in a tribal context through entrepreneurship is an approach for enabling and making them economically and socially viable. This paper aims to highlight entrepreneurship in a specific tribal context and provide insight on some instances or cases relating to women's empowerment. Although there are many initiatives from international organizations and governmental institutions to support women entrepreneurs, especially tribal women, they suffer from isolation as a result of their language and lifestyle differ from the rest of society, which made the growth of their business and ability to compete arduous, and thus affected their ability to make various decisions in their lives. This study examines the pathway to a better understanding of increasing access to entrepreneurship for tribal women in Mayurbhanj district, Odisha.

**Methods:**

The sample size was 111 Santhal tribal women entrepreneurs, and all of them were interviewed using an interview schedule and Focus Group Discussions (FGDs). Two analytical tools were used (a linear regression model to find which dependent variables influence entrepreneurs and the Women's Empowerment Index (WEI) to measure the progress in social and economic opportunities). The respondents was interviewed and asked on the various WEI indicators before and after an entrepreneurship intervention.

**Results and discussion:**

It was observed through the results that the empowerment of women has changed in a positive direction after establishing their work; according to the indicators of the study, the Women's Empowerment Index has changed from 0.61 to 1.26. It was also found that entrepreneurship has a positive and significant impact on women's decision-making within the family, and therefore, it was suggested through research to increase the intervention from the government and related organizations with more initiatives that contribute to the possibility of increasing women's education and their financial ability to open new enterprises.

## 1. Introduction

Women in India are entitled to equal rights with men according to the constitution and laws established by the government. However, rural women do not enjoy the same social and economic freedoms as men, as is the case in urban areas (Kumar, 2013; Agrawal and Khare, 2019). They still rely on men to provide the family's income and spend most of their time on household chores (Datta and Gailey, 2012). Empowering women can serve as a cornerstone for development to achieve Sustainable Development Goals (SDGs), particularly in relation to families, communities, and even nations (Williams et al., 2022). It is the process that equips women with the knowledge and skills necessary to expand their participation, control, and decision-making (Akhter and Cheng, 2020). The trend of making women play an important role has begun to increase in the past two decades, as they started to acquire skills on an equal footing with men and attempted to create their own projects (Kapoor, 2019). Women's engagement in business enhances their capacity in three directions: asset ownership, business cycle ownership and therefore income, and achievement (Andriamahery and Qamruzzaman, 2022). Entrepreneurship has become common among women in India (Datta and Gailey, 2012). It has significantly increased in the past two decades and has become a driving force for the economy (Jahanshahi et al., 2011). Local and non-governmental organizations, as well as governmental organizations, have significantly focused on tribal women, providing numerous initiatives and political decisions, and offering many opportunities to work in poultry and livestock farming, crop cultivation, fruit and vegetable growing, and other types of entrepreneurship. Women's participation from ethnic, tribal, or indigenous backgrounds can contribute to increasing human resources in the country (Sarma, 2014). However, tribal women in India spend most of their time performing their school duties and face numerous challenges such as food insecurity, lack of education, domestic violence, and poor healthcare (Maiti et al., 2005). Therefore, entrepreneurship is not an easy option for them, especially in an environment that suffers from underdevelopment and economic weakness. Lack of funding, education, and difficulty in accessing resources make their business activities less profitable and less effective when compared to other communities.

Studies on women's empowerment through income-generating activities are commonly conducted, however, tribal studies in this area are rare and have not addressed the Santhal tribe located in the Mayurbhanj District, where women have been neglected or excluded from research. To date, there have been no studies on tribal women's leadership in the Mayurbhanj District, which is part of the state of Odisha in India, and no previous statistical studies have been conducted on the significance of the statistical relationship between entrepreneurship, women's empowerment, and decision-making. These are the research gaps that this study will address, making it distinct from other studies. The urgent need to understand how tribal women can continue their businesses is a distant vision for both the government and policy makers, and it should motivate researchers to conduct future research.

This study will contribute to addressing several questions, such as: How can tribal women entrepreneurs succeed in empowering themselves in their communities? Can tribal women really make more household decisions with increased empowerment in entrepreneurship? The relationship between tribal women's empowerment and household decision-making will be explored in order to bring about social change. This study has the potential to positively impact future research by understanding the impact of entrepreneurship on women's empowerment.

The growth of business entrepreneurship among tribal women is very limited and slow, and the study was limited to 111 tribal women, it may have been difficult to conduct an investigation about tribal society due to its special nature. The study was limited to empowering women in decision-making within the family, knowing that there are various other factors such as competence and self-determination and satisfaction with life are not taken into account.

In this perspective, in-depth research is needed to find out whether entrepreneurship increased their decision-making power and made them innovative, risk-taker, and pro-active to continue their business activities or not. Besides, scholars will get to undertake future research on that issue.

## 2. Literature review

Women's empowerment procedures contribute to the improvement of women and are significant change agents (Mehra, 1997). The studies have found that empowerment is an integrated process (Naila, 2005; Akhter and Ward, 2009) that contributes to increasing women's ability to reach great bargaining power when they work outside their homes (Anderson and Eswaran, 2009). Other studies have shown that giving women the opportunity increases their ability to control their income and increases the labor force in general (Field et al., 2021). To restore gender balance, training can create income for women (Creevey and Edgerton, 1997), increase their assets and earnings, and increase the supply of female labor; however, it presents many challenges (Bandiera et al., 2017).

Opportunity is one of the important concepts in entrepreneurship, as researchers consider that entrepreneurs seize opportunities because they have prior knowledge of the labor market, or as a result of developing their social networks and thus knowing the market's need, and because they are distinguished by a set of personal characteristics such as optimism that always drives them as a belief to achieve success in Entrepreneurship (Ardichvili et al., 2003; McMullen et al., 2007). Maslow's theory relies on three dimensions of human needs, including growth, connection, and connection, and that relates to linking the need-satisfaction relationship strongly (Alderfer, 1969).

There are certain tools that aid in promoting the employment of women as Agri-entrepreneurs. These include land ownership, cooperative agriculture, policy implications, and ICT tools, SHG—SME. Regarding the possibilities that can be taken advantage of, Krishi Vigyan Kendras (KVKs), non-governmental organizations, and universities that aim to advance agricultural work are a few examples (Jena et al., 2018). Microcredit is one method of assisting Entrepreneurs in the Odisha regions, and it has a significant positive effect in a number of areas. However, there are some challenges for Entrepreneurs, including the low loan amounts compared to those at the national and regional levels, their reliance on middlemen, and a lack of market access (Rajpal and Tamang, 2021).

Agricultural education in India can help encourage entrepreneurship and follow the best methodologies to succeed in it, as a study by Banerjee et al. (2020) there is a correlation between the desire to work in entrepreneurship, career planning, and agricultural labor capacity. An Indian agricultural university's students were the subjects of this study.

Using the analytical SOWT method, Revelsal and others examined the external and internal factors influencing a small farmer support program called youth agripreneurship development (YAD). It was discovered that both independence and knowledge can be internal factors that have an impact on the community under study, as well as external factors like imposition brought about by the program's resources and threats from both innovation and partnerships. To overcome threats, various strategies have emerged, such as increasing knowledge, using independent technology to increase knowledge, and using both of the knowledge networks (Refiswal et al., 2021).

A one-of-a-kind study by Sarangi et al. (2022) of the impact of immigrants and indigenous entrepreneurs on development in the state of Odisha. Indigenous entrepreneurs have demonstrated a positive impact by increasing overall employment opportunities and facilitating access to immediate employment and adequate livelihoods for marginalized low-skilled workers.

With the help of a structured questionnaire, a study on about 40 entrepreneurs by Agarwal et al. (2021) was carried out across various regions of India (Bungalu, Mumbai, Pune, and roughly 11 other cities). Agricultural entrepreneurs faced several problems, including high costs, shortage of skilled labor, and marketing issues. One of the most important proposals of the study was the creation of a database through which income and expenditures could be recorded for all urban entrepreneurial agricultural projects. In order to establish aquaculture production on a large scale, the study also suggests changing some policies to redistribute land uses and demonstrating greater interest in research collaboration between the public and private sectors. Encouraging the development of arable gardens and landscape design will also help to activate the roles of architects and gardeners.

Joseph Schumpeter gave one of definitions of entrepreneurship and said that every person who works to create a new project and realizes the importance of the opportunity to create a business is considered an entrepreneur (Gill and Ganesh, 2007). But this definition has changed over the years. According to Nkechi et al. (2012), the entrepreneur was described as a creative and innovative person who understands problems and finds appropriate solutions to them. He is the person who performs the entrepreneurial function. Women's entrepreneurship appeared in the late nineties and became important to researchers about 30 years ago. Stephens et al. (2021) mentioned that female entrepreneurs serve the community and bear all the risks in order to gain economic freedom and feel empowered. The research developed the hypothesis based on the concepts of empowerment and entrepreneurship.

## 3. Conceptual framework

This paper used the concepts of women's empowerment and entrepreneurship by following the previous methodologies with the addition of modifications commensurate with the subject of the study and the research area. The term “empowerment” was developed so that it takes on many visions, some of whom mentioned that it is a person's ability to live as he wants (Kabeer, 1999), and it was developed to mean the ability to achieve and obtain resources, and the approach of women's empowerment was widely used by academic researchers and policy makers (Lincoln et al., 2002). It measures income, employment, and the ability of decision-makers within the family; all of these are related to the social and economic dimensions, and all studies found that women are able to achieve satisfaction through their work and provide adequate support for the family.

The process of linking entrepreneurship, empowerment, and decision-making is not easy because the concept of entrepreneurship has many dimensions and is difficult to measure. Increasing profits and the concept of empowerment must be linked to the development of entrepreneurship, as more achievements at the level of entrepreneurship will generate more profits and thus be reflected in empowerment and the decision-making process. As the empowerment tool is the main key to the success of setting up any enterprise, it is weighted by material procurement, managing the activities of a business, labor hire, price setting, and selling products. For family issues, family life education, household maintenance, savings and expenditures balance, medical care, coping mechanisms, arranged marriage, etc. are all considered. Training participation, ability to visit friends, families, and others, ability to use public transportation, and women's use of media, etc. are measured for mobility. Knowledge is weighted by working rights knowledge; education is important, as are sanitation, pure drinking water, food preservation, and treatment.

**Figure 1:**
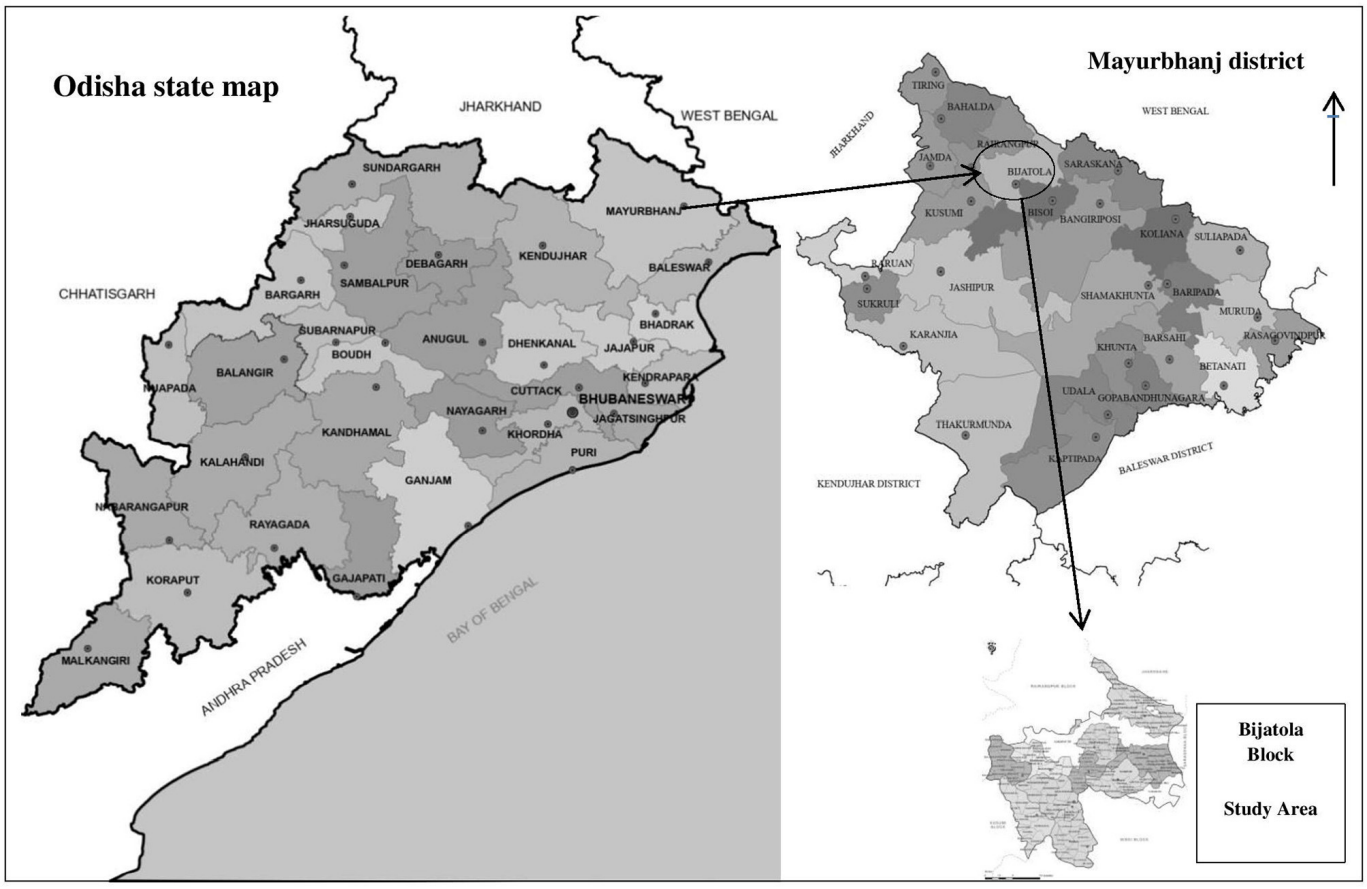
Conceptual framework.

Figure 1 shows the available opportunities for entrepreneurship. In this approach, we find that entrepreneurship makes women active, risk-taking, innovative, and faces many challenges to reach a successful and profitable business, It also makes women confident in making individual decisions and feels empowered.

This research doesn't just add a new definition and meaning for “entrepreneurship” and “women's empowerment,” it also provides new tools and indicators that may be used to measure the importance of tribal women in India.

The study is an analysis of reality and a translation of what exists for tribal entrepreneurship, and its aims are as follows: (1) Find the factors of entrepreneurship for women in the studied area (empowerment and decision making). (2) To estimate the relationship between empowerment and entrepreneurship. (3) To know the basics of reaching decisions within the family of tribal women.

## 4. Materials and methods

**Figure 2:**
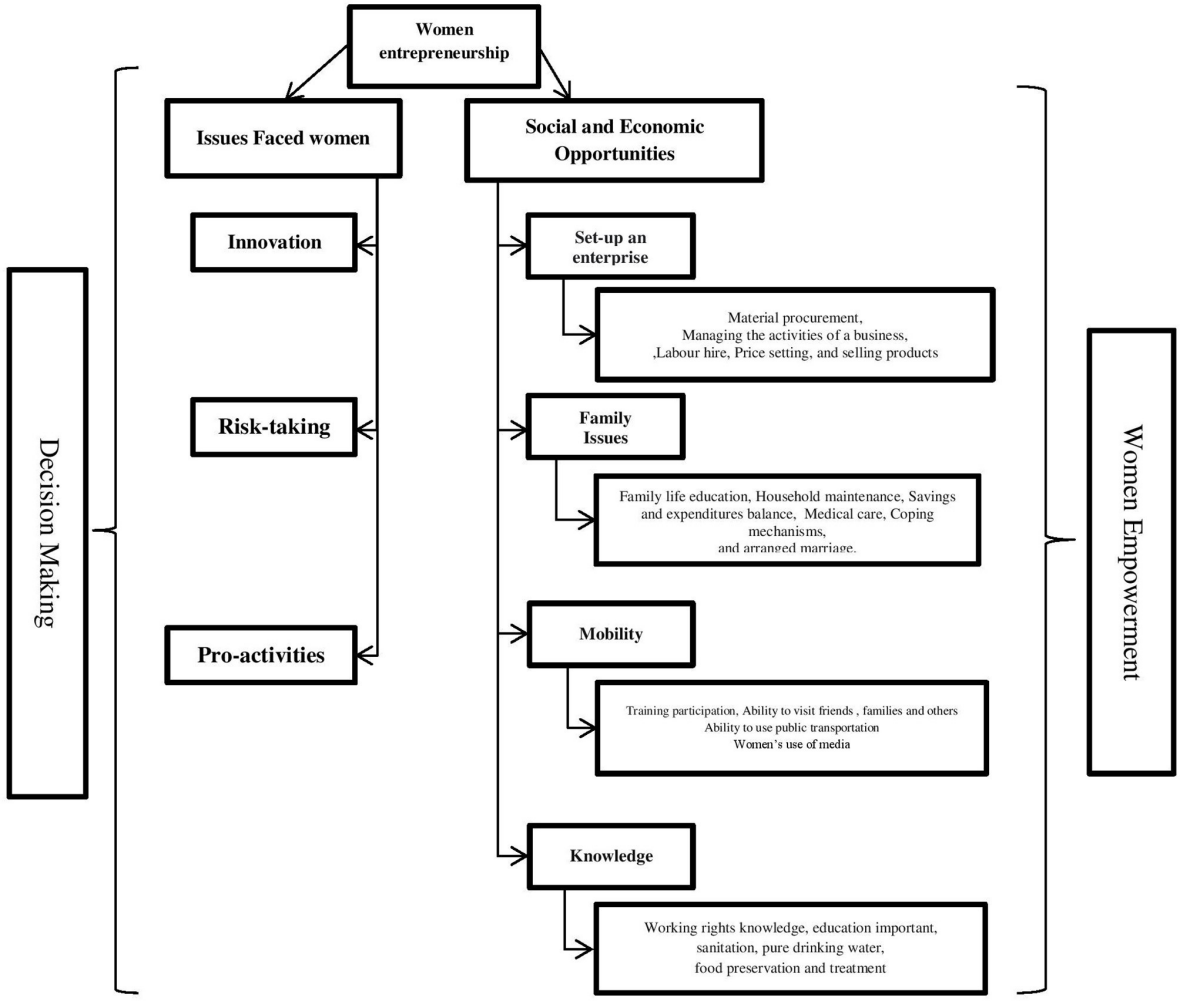
Location map of the study areas in India.

The study was conducted in Mayurbhanj District, which is located in Odisha state, India. The data were collected from Bijatola block, where Santhal tribes constitute about 77% of its total population (Mohanty, 2017). A multi-stage sampling technique was used to select sample farm households in the study area. The first stage was to select 13 villages in which Santhal tribes are present. In order to identify the women entrepreneurs in these villages, the second stage was to contact key informants in each village; thereafter, simple random sampling was used to select 111 women entrepreneurs. The interview schedule has been designed based on the conceptual framework to conduct face-to-face interviews and focus group discussions (FGDs) with 20 participants in each village to collect some primary data information and reach the research objective (Figure 2).

Linear regression model used to identify the influencing factors, where entrepreneurship was dependent variable (Y), and the independent variables were as follows: age (X1), family size (X2), literacy (X3), membership with NGOs (X4), number of training hours (X5), and personal business revenue (X6). Here is the formula that was used:


$$\begin{array}{l} Y_{i}=a+\beta_{1}X_{1}+\beta_{2}X_{2}+\beta_{3}X_{3}+B_{4}X_{4}+\beta_{5}X_{5}+\beta_{6}X_{6}+U_{i} \end{array}$$

Here, the change in one of the regressions is measured by the marginal effect (ME). Marginal effects assess discrete change as a method of predicting probability changes when independent variables are present. In this aspect, six social and economic variables—including age, education, number of training courses, business revenue, family size, and membership with NGOs—are taken into consideration to assess a reliable estimation of the extent of change in entrepreneurship. When all other variables were held constant at their means, the marginal effects demonstrated how the identified factors changed from 0 to 1.

Women Empowerment Index (WEI) used considering before and after the entrepreneurship situation. After the entrepreneurship situation. Non-entrepreneurial situations of the selected tribal women was the best for baseline data. This is based on choosing a base or end line for entrepreneurship, which ranges from 5 to 7 years of experience. Experts, relevant individuals, and researchers discussed the choice of 20 indicators for women's empowerment, which are: five for setting up an enterprise, six for family issues, four for mobility, and five for knowledge, which were used in the study, so that the highest value is 2 (women are leaders in decision-making) and is in a state of empowerment, and the lowest value is 0, which indicates men's control over decision-making is in a state of disempowerment.

Women Empowerment Index (WEI) was given in equations (Bose et al., 2009) as the following:


$$\begin{array}{l} WEI_{i}\;=\;\frac{\sum_{i=1}^{n}xi}{n}\; \end{array}$$

Where women are not empowered when the WEI is ≤1, and they are empowered when the WEI is more than 1, and *n* is based on the number of dimensions under each indicator, such as family issues, n is equal to 6.

Women's entrepreneurship was evaluated on the basis of three components, including innovation, risk-taking, and pro-activities, which were further evaluated by nine items, three of each. While the four components of the decision-making process were used to understand the level of empowerment among tribal women entrepreneurs, Confirmatory Factor Analysis (CFA) was used to validate the latent construct's fitness, reliability, and validity. Composite reliability testing was also done to guarantee the data's internal consistency.

## 5. Results and discussion

Entrepreneurs were divided according to age into several groups, and the highest percentage was (67.6) between 35 and 45 years old, and the percentage of women who could write and read was about 14.41%. This low percentage may be due to the difficulty of reaching tribal women, or it may be the result of the educational curricula (Mohanty, 2017), or caused by the poor financial situation of the family and their inability to go to school, because they work in collecting forest products for their livelihood (Tudu and Das, 2022). A study by Haddad et al. (2016) indicates that education is factor that can influence choice behavior and profoundly affect personal development. About 59.5% of the households in the research sample were found to have households with an average number of 4–6 members, which is higher than the national average household size based on Global Data 2021. It was also noted from Table 1 that a number of women in the sample have personal business revenue that exceeds 10,000 rupees, which is about 80.2% of the total. Women indicated that there is a priority in spending, and the importance is different, as spending on food is followed by spending on education for children, and then clothes. Table 1 shows that families live in *Pucca* houses with mud walls and tin roofs (85.6%), and some women live in houses with bamboo roofs. The sanitary system is not good, and 9% of the women houses (with half of the building) is not used. Socioeconomic status of tribal women has studied by a few researches (Subramanian et al., 2006; Mungreiphy and Kapoor, 2010); to find out their sustainable livelihood status, Kaushal and Kala (2004) conducted a study and measured their livelihood disparities.

**Table 1:** Characteristics of women entrepreneurs in the sample (*n* = 111).

**Social characteristics**		**Frequencies**	**%**
Age group	Women up to 35 years old	15	13.5
	Women from 35 to 45 years old	75	67.6
	Women above 45 years old	21	18.9
Family size	Small families (1–3 members)	22	19.8
	Medium families (4–6 members)	66	59.5
	Large families (7 and above)	23	20.7
Literacy rate	Women with literacy	16	14.41
**Economic characteristics**		**-**	**-**
Personal business revenue	Below Rs. 10,000	22	19.8
	From Rs. 10,000 to 25,000	70	63.1
	Above Rs. 25,000	19	17.1
Household expenditure based on the important	Children education	20	18
	Food	70	63.11
	Clothes	12	10.8
	Medicine	5	4.5
	Others	4	3.6
Type of the house	*Pucca*	95	85.6
	*Katcha*	10	9
	Half building	6	5.4

Own research.

Four of the explanatory variables that contributed to taking up entrepreneurship, success, increase income (literacy, membership with NGOs, number of trainings, and personal business revenue). Literacy and the number of training sessions helped them make more informed decisions in their enterprise by directly updating their knowledge and increasing their capacity for understanding. They were able to increase their confidence and lessen their economic vulnerability due to higher personal business revenue and membership with NGOs.

Age is one of the variables positively affecting entrepreneurship, but in the studied case, age was not significantly associated with entrepreneurship, and this may be because the majority of the research sample were middle-aged women (from 35 to 45 years old) and they were not very interested in new projects but rather in developing their traditional projects. Table 2 shows that the size of the family contributed negatively to entrepreneurship, which may be a result of the increased work that women do at home and the lack of time during which they can control the project. It may also be due to a lack of money after securing the various needs of the family.

**Table 2:** Maximum Likelihood Estimation (MLE) results.

**Explanatory variables**	**Coefficient**	**Standard error**	***Z*-statistic**	**Probability**
Age (X1)	0.061	0.312	0.71	−0.0412
Family size (X2)	−0.045	0.111	−0.39	−0.221
Literacy (X3)	1.062^**^	0.323	4.50	0.614
Membership with NGOs (X4)	2.160^**^	0.361	8.14	1.735
Number of training (X5)	2.537^**^	0.482	5.91	1.435
Personal business revenue (X6)	0.00041^*^	0.00026	2.11	0.001
Constant	−7.553	1.257	−2.34	−12.131
Pseudo *R*^2^	0.7518			
LR Chi square	512.22			
Log likelihood	−37.321176			

^*^Significant at 5% level.

^**^Significant at 1% level.

According to Table 3, the chance of starting a project increases by 0.3% as age increases 1 year. Other variables also had the same positive effect, such as changes in education, the number of trainings, and personal income, which increased by 51, 7, and 0.04%, respectively. As for the membership with organizations, it was the only one with a positive and significant effect, at 71%. Several studies have been conducted on tribals in Mayurbhanj District, Odisha, on their traditional knowledge (Panda et al., 2011), their education statue (Behera, 2015), the impact of women participants in Self-Help Groups (SHGs) microfinance programs (Rajpal and Tamang, 2014; Rajpal, 2016), and the constraints faced by them to become entrepreneurs (Mohapatra et al., 2012).

**Table 3:** Marginal probability results.

**Explanatory variables**	**Standard error**	***Z*-statistic**	**Probability**	**Marginal effect**
Age (X1)	0.022	0.71	0.421	0.0031
Family size (X2)	0.0412	−0.39	0.564	−0.013
Literacy (X3)	0.071	4.50	0.000	0.514^**^
Membership with NGOs (X4)	0.05103	8.14	0.000	0.711^**^
Number of training (X5)	0.0776	5.91	0.000	0.513^**^
Personal business revenue (X6)	0.00014	2.11	0.017	0.00041^*^

^*^Significant at 5% level.

^**^Significant at 1% level.

The women's empowerment index often shows the extent to which women are able to take a set of actions and decisions as a result of entrepreneurship, as they can achieve financial independence and become more powerful than before in many ways, such as making the decision to marry and children, control living expenses, and attend social festivals. With some exceptions for men, which give men the ability to make decisions, such as recruitment processes for labor to assist in entrepreneurial activities. We note from Table 4 that women entrepreneurs have been empowered to a greater extent compared to before entrepreneurship, and the women's empowerment index reached 1.26. It is noted from the table that among tribal women, women have been empowered in terms of mobility (1.32), followed by decisions related to the setup of an enterprise (1.30), then decisions related to family issues (1.28), and finally knowledge (1.14).

**Table 4:** Results of Women Empowerment Index.

**Indicators**	**WEI before entrepreneurship**	**WEI after entrepreneurship**
Set-up an enterprise	0.55	1.30
Material procurement	0.28	1.15
Managing the activities of a business	0.67	1.27
Labor hire	0.25	1.17
Price setting	0.71	1.49
Selling products	0.82	1.44
Family issues	0.67	1.28
Family life education	0.73	1.13
Household maintenance	0.77	1.33
Savings and expenditures balance	1.04	1.55
Medical care	0.41	1.29
Coping mechanisms	0.30	1.16
Arranged marriage	0.76	1.25
Mobility	0.61	1.32
Training participation	0.67	1.44
Ability to visit friends, families, and others	0.51	1.06
Ability to use public transportation	0.66	1.35
Women's social festival attending	0.95	1.43
Knowledge	0.62	1.14
Working rights knowledge	0.51	1.06
Education important	0.41	1.05
Sanitation	0.56	1.08
Pure drinking water	0.66	1.34
Food preservation and treatment	0.96	1.15
Overall WEI	0.61	1.26

Own research.

To verify the reliability of the data, a set of tests, including Cronbach's alpha, which gave a low value, was run on the questionnaire and groups of questions (Table 5).

**Table 5:** Reliability and validity tests results.

**Construct**	**Concept**	**Cronbach's alpha**	**Reliability test result**
Entrepreneurship	Empowerment	0.501	0.146
	Decision making	0.289	0.079

Own research.

It turned out that the questions were not sufficient to know the extent of empowerment of tribal women and needed more focus, while the degree of reliability was acceptable to some extent in the study area.

The rate of women's empowerment was not as high as expected, as the results showed that it was at a low level. The results from Table 6 show that entrepreneurship increased women's ability to make decisions related to innovation, risk, and pro-activities, but despite all that, it demanded more attention from them. The implication was that tribal women gained empowerment and an enhanced intra-household decision-making process as a result of entrepreneurship.

**Table 6:** Descriptive statistics of women's decision making.

**Concept**	**Indicators**	**β**	**Mean**	**Standard deviation**	***t*-value**	***P*-value**
Decision making	Innovation: Launch new product	0.311	1.23	0.014	21.05	0.000
	Risk-taking: Goal oriented	0.308	1.16	0.015	20.86	0.000
	Pro-activities: Initiative oriented	0.281	1.2	0.018	22.67	0.000

Own research.

The results show that tribal women entrepreneurs have contributed to setting up their businesses by more than 50%, followed by decisions related to transportation by 33%, while decisions related to the family are low. Therefore, the results in general are that entrepreneurship has helped empower women, which has led to a sense of creativity and increased decision-making.

## 6. Conclusion

One of the main objectives of this paper was to determine whether or not entrepreneurship inspired women's empowerment in tribal society, taking into account the relationship between entrepreneurship, women's empowerment, and decision-making. The study adds to the existing body of knowledge on how entrepreneurship promotes tribal women's identity and recognition in their communities. The findings show that four of the explanatory variables that contributed to making women entrepreneurs in the tribal community (literacy, membership with NGOs, training, and personal business revenue) helped increase the empowerment of women within their families and society, despite many social and cultural challenges and the difficulty of accessing resources as a result of preserving their mother tongue and being introverted. There are a set of limitations to the study, including the inability to cover more than 111 tribal women as a result of the difficulty of conducting investigations in the tribal community. There were no other dimensions, such as life satisfaction and self-determination for women entrepreneurs, that were not taken into consideration. The results of this study should be used by the government and relevant agencies to formulate special policies aimed at encouraging tribal women entrepreneurs in India by increasing the number of training and education opportunities commensurate with the privacy of these communities. The study suggests more loans and an increase in awareness of the importance of business and its potential, as well as the need for future research that includes other factors to find out how to empower women through entrepreneurship more, which leads to increased economic opportunities, participation, and educational attainment.

## Data availability statement

The raw data supporting the conclusions of this article will be made available by the authors, without undue reservation.

## Ethics statement

Ethical review and approval was not required for the study on human participants in accordance with the local legislation and institutional requirements. Written informed consent for participation was not required for this study in accordance with the national legislation and the institutional requirements.

## Author contributions

All authors listed have made a substantial, direct, and intellectual contribution to the work and approved it for publication.
